# Discontinuous Doppler‐Derived Intrarenal Venous Flow Is a Predictor of Impaired Exercise Capacity Associated With Poor Prognosis in Patients With Acute Coronary Syndrome

**DOI:** 10.1111/echo.70192

**Published:** 2025-06-01

**Authors:** Kenji Masada, Kento Fujita, Misato Saito, Masashi Kodama, Yoji Sumimoto, Takashi Shimonaga, Haruyuki Kinoshita, Hiroshi Sugino

**Affiliations:** ^1^ Department of Cardiology National Hospital Organization Kure Medical Center Kure Japan

**Keywords:** acute coronary syndrome, cardiopulmonary exercise testing, discontinuous Doppler‐derived intrarenal venous flow, exercise capacity, peak early diastolic mitral inflow velocity to early diastolic velocity ratio

## Abstract

**Background:**

Doppler‐derived intrarenal venous flow (IRVF) has recently been used to assess renal congestion and intrarenal hemodynamics. Although several reports suggest that IRVF is useful for predicting the prognosis of patients with heart failure (HF), information is sparse for patients with acute coronary syndrome (ACS). Therefore, we performed a study to investigate the relationship between IRVF and peak oxygen consumption (VO_2_), which is associated with prognosis in patients with ACS.

**Methods and Results:**

We enrolled 80 patients with ACS. The prevalence of discontinuous IRVF (DIRVF) was higher in patients with peak VO_2_ less than the group median (13.2 mL/min/kg; 38% vs. 8%, *p* = 0.003). Multivariable logistic regression analyses indicated that DIRVF was the only independent predictor of peak VO_2_ <12 mL/min/kg (odds ratio 6.33, 95% confidence interval [CI] 1.28−31.1, *p* = 0.02). Median follow‐up was 366 days [189−513 days]. The occurrence of composite endpoints, including death from cardiovascular disease and unplanned hospitalization for HF, was significantly higher in patients with DIRVF than in those with continuous IRVF (*p =* 0.001). Moreover, according to receiver operating characteristic curves, the area under the curve obtained with basic clinical variables (age, sex, and log N‐terminal pro‐B‐type‐natriuretic peptide) was 0.72 (95% CI 0.60−0.83); this increased significantly to 0.84 (95% CI 0.75−0.93) when peak early diastolic mitral inflow velocity to early diastolic velocity ratio (E/e’) and DIRVF were added (*p* = 0.007).

**Conclusions:**

DIRVF predicts impaired exercise capacity, which is associated with poor prognosis, in patients with ACS.

## Graphical abstract

DIRVF was an independent predictor of impaired exercise capacity and poor prognosis in patients with ACS. Addition of DIRVF to a clinical variable‐based model improved its predictive ability for impaired exercise capacity in patients with ACS.

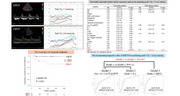

**Abbreviations**
ACS: acute coronary syndrome
CI: confidence interval
DIRVF: discontinuous Doppler‐derived intrarenal venous flow
HF: heart failure
IRVF: Doppler‐derived intrarenal venous flow
VO_2_
: oxygen consumption

## 1 Introduction

Doppler‐derived intrarenal venous flow (IRVF) has recently been used to assess renal congestion and intrarenal hemodynamics [1, 2, 3, 4, 5, 6, 7]. Renal congestion occurs mainly in the renal parenchyma and is accompanied by an increase in renal interstitial pressure [8]. Changes in parenchymal conditions can compress blood vessels in the renal parenchyma and reduce vascular compliance, both of which increase central venous pressure via increased resistance. Consequent changes in vessel shape and function can lead to transient and cardiac cycle‐dependent stasis of renal venous flow and changes in IRVF patterns.

This interdependent dysfunction of the heart and kidneys, termed cardiorenal syndrome, is important in heart failure (HF) pathophysiology [9]; indeed, several reports suggest that IRVF can predict the prognosis of patients with HF [1, 2, 3, 4, 5, 6]. However, no studies have reported the correlation between IRVF and the prognosis of patients with acute coronary syndrome (ACS).

Reduced exercise capacity is an important predictor of poor prognosis and disability in patients with coronary artery disease, including ACS [10, 11, 12]. Peak oxygen consumption (VO_2_) assessed by cardiopulmonary exercise testing (CPET) is a widely used and well‐established parameter for measuring exercise capacity [13].

Previous studies suggest a relationship between peak VO_2_ and echocardiography‐determined parameters in patients with ACS [12, 14, 15]; especially, left ventricular diastolic dysfunction represented by peak early diastolic mitral inflow velocity to early diastolic velocity ratio (E/e’) is associated with lower peak VO_2_.

However, for patients with ACS, the relationship between IRVF patterns and peak VO_2_ is not well understood. As IRVF reflects right‐sided heart hemodynamics [6, 7], we hypothesized that, like E/e’, IRVF patterns would be closely related to peak VO_2_. To test this hypothesis, we investigated the relationship between IRVF patterns and peak VO_2_, which is associated with prognosis, in patients with ACS.

## 2 Methods

### 2.1 Study Population

Ninety‐six consecutive patients with ACS who underwent percutaneous coronary intervention at National Hospital Organization Kure Medical Center between January 2023 and September 2024 were assessed for eligibility; those who did not meet exclusion criteria were prospectively enrolled. ACS was diagnosed according to current guidelines [16, 17]. The exclusion criteria were as follows: hemodynamically significant valvular disease, atrial fibrillation, presence of a pacemaker, inability to exercise, unacceptable image quality, and death before testing. The research protocol was approved by our institutional ethics committee. All patients provided written informed consent.

### 2.2 Echocardiography

An experienced clinician (Y.S.) without knowledge of the patients’ clinical status performed both echocardiography and renal Doppler ultrasonography using a Vivid E9 ultrasound system with a sector transducer frequency range of 2.5―5.0 MHz transducer (GE Vingmed Ultrasound, Horten, Norway). All imaging data were digitized and saved on an optical disc for off‐line analysis (Echo Pac software version 112, GE Vingmed Ultrasound). All echocardiographic measurements were taken according to the recommendations of the American Society of Echocardiography [18]. All parameters were measured in triplicate and averaged. Standard two‐dimensional and Doppler blood flow recordings were performed using standard methods [18].

IRVF was recorded in the right kidney with the patient in the left lateral decubitus position. Color Doppler images were used to determine interlobar vessels, and the sample volume was set based on the color Doppler signals derived from interlobar veins.

In accordance with previous reports [3, 4, 5, 6, 7], Doppler waveforms of IRVF were divided into three flow patterns: continuous, biphasic discontinuous, and monophasic discontinuous. Discontinuous flow types had more than one phase with zero velocity.

The IRVF patterns were jointly determined by Y.S. and M.T. (an experienced sonographer). We defined biphasic and monophasic IRVF as discontinuous IRVF (DIRVF) in relation to continuous IRVF (CIRVF).

### 2.3 CPET

Patients performed exercise testing with concurrent ventilator expired gas analysis on an upright bicycle ergometer. Patients pedaled at a constant speed (50 rotations/min), starting with an initial workload of 10 W for 3 min, which was then increased by 10 W/min (ramp protocol). During the test, a 12‐lead electrocardiogram and heart rate were continuously monitored, and blood pressure was measured every minute. The test was terminated upon signs of physical exertion or severe distress. We measured VO_2_, carbon dioxide production, and other common ventilator parameters on a breath‐by‐breath basis using gas analysis (MINATO 280S; Minato Ikagaku, Osaka, Japan).

**FIGURE 1:**
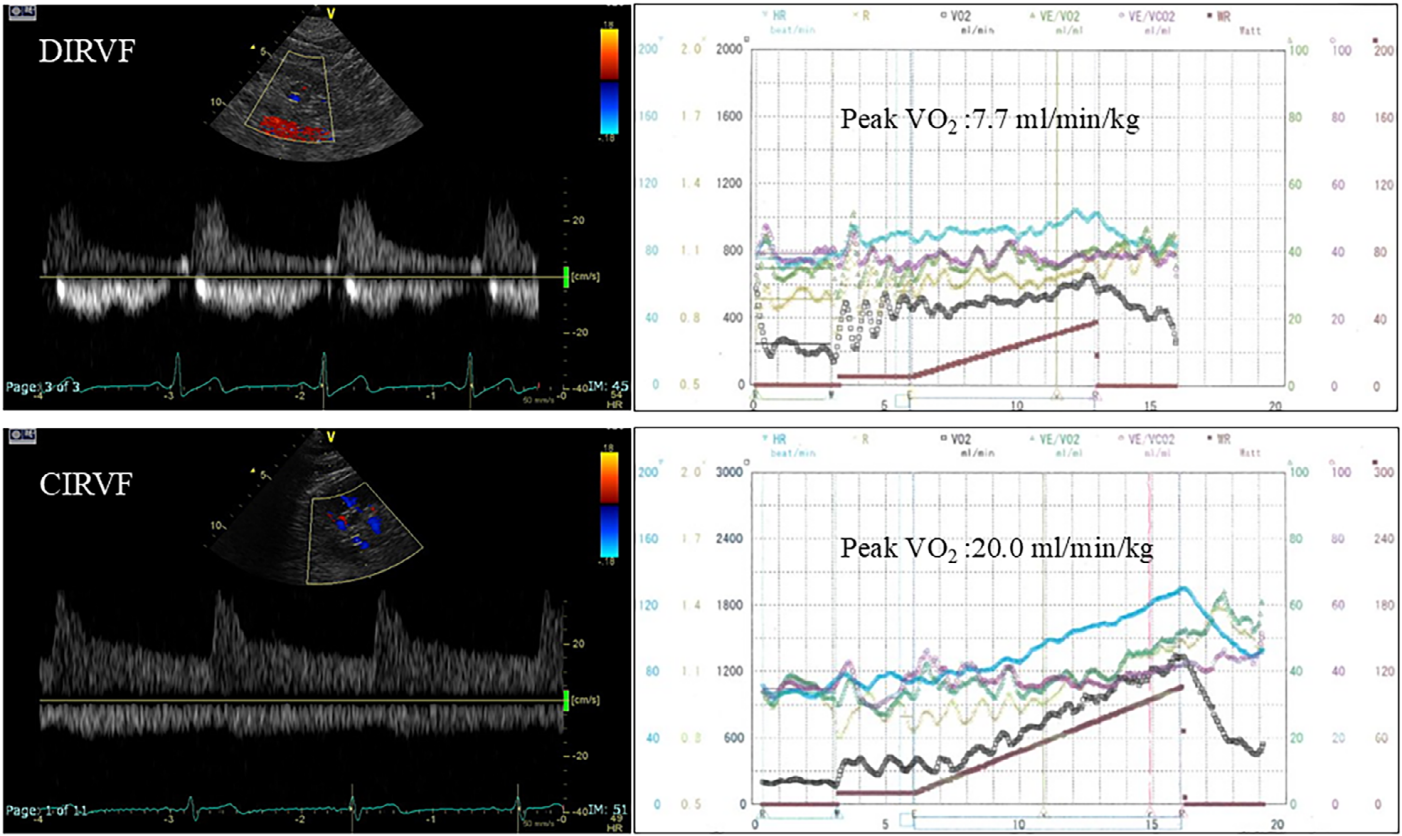
(Left) Continuous (lower row) and discontinuous (upper row) Doppler‐derived intrarenal venous flow. (Right) Corresponding findings of cardiopulmonary exercise testing. CIRVF, continuous Doppler‐derived intrarenal venous flow; DIRVF, discontinuous Doppler‐derived intrarenal venous flow.

We defined impaired exercise capacity as peak VO_2_ < 12 mL/min/kg in the presence of β‐blockers, in accordance with a previous report [19]. All patients underwent both a transthoracic echocardiogram and CPET in the 3 days before hospital discharge (Figure 1).

### 2.4 Clinical Outcome

Clinical outcomes, including death from cardiovascular disease and unplanned hospitalization for HF, were recorded.

### 2.5 Statistical Analysis

Continuous variables are expressed as the mean ± standard deviation, median (25th and 75th percentiles), or number (proportion, %). Discrete variables and proportions were compared using a χ^2^ analysis or Fisher's exact test. Comparisons between two groups were performed using a Student's unpaired *t*‐test or a Mann−Whitney *U* test.

Independent predictors of impaired exercise capacity (peak VO_2_ < 12 mL/min/kg) and DIRVF were identified using multivariable logistic regression analyses. Variables identified as statistically significant in univariable analyses were included in the final model.

To evaluate the incremental value of DIRVF and E/e’ for predicting peak VO_2_ < 12 mL/min/kg in addition to basic clinical variables (age, sex, and log N‐terminal pro‐B‐type‐natriuretic peptide [NT‐proBNP]), receiver operating characteristic curves (ROCs) were plotted. Areas under the curve (AUCs) were compared using Delong test.

Cumulative incidences of clinical events were calculated using the Kaplan−Meier method, and differences in event rates between two groups were compared using the log‐rank test.

A *p* value < 0.05 was considered statistically significant. Data were analyzed using R software (R version 3.4.0, R Foundation for Statistical Computing, Vienna, Austria).

## 3 Results

Among 96 consecutive patients with ACS, 16 patients were excluded for the following reasons: hemodynamically significant valvular disease (*n* = 1), atrial fibrillation (*n* = 2), presence of a pacemaker (*n* = 1), inability to exercise (*n* = 3), unacceptable image quality (*n* = 3), and death before transthoracic echocardiogram testing or CPET (*n* = 6). Therefore, we enrolled 80 patients and divided them into two groups according to a median peak VO_2_ of 13.2 mL/min/kg. Table 1 provides a summary of the baseline demographic and clinical characteristics of 80 patients. Compared with patients with peak VO_2_ ≧ 13.2 mL/min/kg, those with peak VO_2_ < 13.2 mL/min/kg were older (72 ± 11 years vs. 66 ± 11 years, *p* = 0.02), and more likely to have hypertension (93% vs. 75%, *p* = 0.04), diabetes (48% vs. 23%, *p* < 0.001), and to take calcium channel blockers (30% vs. 3%, *p* = 0.002) or diuretics (73% vs. 43%, *p* = 0.02). Moreover, patients with peak VO_2_ < 13.2 mL/min/kg had higher creatinine (1.0 [0.9–1.3] mg/dL vs. 1.0 [0.8–1.1] mg/dL, *p* = 0.03), hemoglobin A_1_c (6.6% ± 1.0% vs. 6.1% ± 0.7 %, *p* = 0.009), and log NT‐proBNP (6.7 ± 1.4 vs. 6.2 ± 1.3, *p* = 0.008) levels than those with peak VO_2_ ≧ 13.2 mL/min/kg.

**TABLE 1:** Baseline clinical characteristics.

	All	Peak VO_2_ ≧ 13.2	Peak VO_2_ < 13.2	
Valiables	(*n* = 80)	(*n* = 40)	(*n* = 40)	*p* value
Age (years)	69 ± 12	66 ± 11	72 ± 11	0.02
Males (%)	60 (75)	32 (80)	28 (70)	0.44
BSA (m^2^)	1.7 ± 0.2	1.7 ± 0.2	1.7 ± 0.2	0.33
BMI (kg/m^2^)	24.3 ± 3.9	23.7 ± 3.4	25.0 ± 4.3	0.15
STEMI, *n* (%)	59 (74)	29 (73)	30 (75)	1.0
Multi‐vessel disease, *n* (%)	28 (35)	11 (28)	17 (43)	0.24
Curprit lesion				
RCA, *n* (%)	29 (36)	11 (28)	18 (45)	0.08
LAD, *n* (%)	44 (55)	24 (60)	20 (50)	0.37
LCX, *n* (%)	7 (9)	5 (13)	2 (5)	0.57
Hypertension, *n* (%)	67 (84)	30 (75)	37 (93)	0.04
Diabetes, *n* (%)	28 (35)	9 (23)	19 (48)	<0.001
Hypercholesterolemia, *n* (%)	50 (63)	24 (60)	26 (65)	0.43
Smoking, *n* (%)	41 (51)	21 (53)	20 (50)	0.38
Hemoglobin (g/dL)	12.8 ± 1.9	12.9 ± 2.0	12.8 ± 1.9	0.89
Creatinine (mg/dL)	1.0 (0.9–1.2)	1.0 (0.8–1.1)	1.0 (0.9–1.3)	0.03
Aspartate transaminase (IU/L)	24 (16–32)	24 (18–32)	23 (16–32)	0.88
γ‐glutamy transpeptidase, IU/L	29 (18–58)	34 (21–63)	25 (16–46)	0.60
Maximum creatine kinase (IU/L)	1,617 (492–3,187)	956 (430–2,626)	1,959 (1,061–3,465)	0.11
Hemoglobin A1c (%)	6.4 ± 0.9	6.1 ± 0.7	6.6 ± 1.0	0.009
Log NT‐proBNP	6.5 ± 1.4	6.2 ± 1.3	6.7 ± 1.4	0.008
Medications, *n* (%)				
β‐blockers	76 (95)	37 (93)	39 (98)	0.22
ACE inhibitors/ARAs	75 (94)	36 (90)	39 (98)	0.37
Calcium channel blockers	13 (16)	1 (3)	12 (30)	0.002
Duretics	46 (58)	17 (43)	29 (73)	0.02

*Note*: Values are presented as mean ± SD, median (25th and 75th percentiles), or *n* (%);

Abbreviations: ACE, angiotensin‐converting enzyme; ARA, angiotensin receptor antagonist; BMI, body mass index; BSA, body surface area; LAD, left anterior descending artery; LCX, left circumflex artery; NT‐proBNP, N‐terminal pro‐B‐type‐natriuretic peptide; RCA, right coronary artery; STEMI, ST elevation myocardial infarction.

### 3.1 Echocardiography

The echocardiography parameters of the patients are listed in Table 2. Patients with peak VO_2_ < 13.2 mL/min/kg had larger or higher values than those with peak VO_2_ ≧ 1 3.2 mL/min/kg for the following parameters: interventricular septum thickness (1.0 ± 0.2 cm vs. 0.9 ± 0.2 cm, *p* = 0.02), left ventricular global longitudinal strain (LVGLS) (−12.2% ± 3.8% vs. ―14.0% ± 3.4%, *p* = 0.03), E/e’ (13.2 [9.9−16.8] vs. 9.4 [7.3−11.7], *p* = 0.005), and DIRVF (38% vs. 8%, *p* = 0.003). By contrast, patients with peak VO_2_ < 13.2 mL/min/kg had smaller or lower values than those with peak VO_2_ ≧13.2 mL/min/kg for the following parameters: left ventricular ejection fraction (49.3% ± 11.0% vs. 54.1% ± 9.5%, *p* = 0.04), e’ (5.2 ± 1.4 cm/s vs. 6.4 ± 2.2 cm/s, *p* = 0.004), and tricuspid annular plane systolic excursion (TAPSE) (1.7 ± 0.4 cm vs. 2.0 ± 0.4 cm, *p* = 0.004).

**TABLE 2:** Echocardiography and cardiopulmonary exercise testing.

	All	Peak VO_2_ ≧ 13.2	Peak VO_2_ < 13.2	
Valiables	(*n* = 80)	(*n* = 40)	(*n* = 40)	*p* value
Echocardiography				
LAD (cm)	4.0 ± 0.5	3.9 ± 0.4	4.1 ± 0.6	0.12
IVS (cm)	1.0 ± 0.2	0.9 ± 0.2	1.0 ± 0.2	0.02
PW (cm)	0.9 ± 0.1	0.9 ± 0.1	1.0 ± 0.1	0.13
LVDd (cm)	4.9 ± 0.5	4.9 ± 0.5	4.9 ± 0.6	0.93
LVDs (cm)	3.6 ± 0.6	3.6 ± 0.6	3.7 ± 0.6	0.31
LVEF (%)	51.7 ± 10.5	54.1 ± 9.5	49.3 ± 11.0	0.04
LVGLS (%)	‐ 13.1 ± 3.7	‐ 14.0 ± 3.4	‐ 12.2 ± 3.8	0.03
E wave, cm/s	60.9 ± 18.2	57.8 ± 16.5	64.1 ± 19.5	0.12
A wave (cm/s)	77.5 ± 20.2	73.6 ± 19.0	81.6 ± 20.8	0.08
E/A	0.73 (0.64–0.93)	0.71 (0.66–0.90)	0.74 (0.64–0.99)	0.48
e' (cm/s)	5.8 ± 1.9	6.4 ± 2.2	5.2 ± 1.4	0.004
E/e'	11.0 (8.0–14.8)	9.4 (7.3–11.7)	13.2 (9.9–16.8)	0.005
IVC diameter (cm)	14.0 ± 3.5	14.1 ± 3.5	13.9 ± 3.6	0.85
TRV (m/s)	2.2 (1.8–2.5)	2.2 (2.0–2.4)	2.2 (0.0–2.5)	0.45
TAPSE (cm)	1.8 ± 0.4	2.0 ± 0.4	1.7 ± 0.4	0.004
RV s' (cm/s)	11.0 ± 3.1	11.5 ± 3.1	10.6 ± 3.1	0.20
RV e' (cm/s)	6.6 ± 2.4	6.9 ± 2.7	6.3 ± 2.1	0.30
FAC (%)	39.4 ± 7.6	39.9 ± 6.0	38.8 ± 9.2	0.56
DIRVF, *n* (%)	18 (23)	3 (8)	15 (38)	0.003
Cardiopulmonary exercise testing				
Rest VO_2_, mL/min/kg	3.3 ± 0.6	3.5 ± 0.5	3.2 ± 0.6	0.007
Anaerobic threshold (mL/min/kg)	9.0 ± 1.7	10.2 ± 1.2	7.6 ± 1.1	<0.001
Peak VO_2_ (mL/min/kg)	12.8 ± 3.9	16.1 ± 2.0	9.6 ± 2.2	<0.001
Rest HR (bpm)	68 ± 10	69 ± 9	67 ± 11	0.34
Peak HR, bpm	105 ± 20	118 ± 15	92 ± 15	<0.001
Rest systolic BP (mmHg)	117 ± 21	116 ± 20	118 ± 22	0.66
Peak systolic BP (mmHg)	159 ± 30	169 ± 28	148 ± 27	<0.001
VE/VCO_2_ slope	37.7 ± 10.1	33.9 ± 5.7	41.6 ± 12.2	<0.001
Peak load (watt)	73 ± 29	92 ± 23	52 ± 22	<0.001

Abbreviations: A wave, late mitral diastolic inflow velocity; BP, blood pressure; DIRVF, discontinuous Doppler‐derived intrarenal venous flow; E wave, early mitral diastolic inflow velocity; e', mitral relaxation velocity; E/A, early to late mitral inflow velocities ratio; E/e', mitral inflow to mitral relaxation velocity ratio; FAC, fractional area change; HR, heart rate; IVC, inferior vena cava; IVS, interventricular septum; LAD, left arterial dimension; LVDd, left ventricular dimension in diastole; LVDs, left ventricular dimension in systole; LVEF, left ventricular ejection fraction; LVGLS, left ventricular global longitudinal strain; PW, posterior wall; RV e', early diastolic annular tissue velocity of the lateral tricuspid annulus; RV s', systolic annular tissue velocity of the lateral tricuspid annulus; TAPSE, tricuspid annular plane systolic excursion; TRV, tricuspid regurgitation velocity; VE/VCO_2_ slope, relationship between minute ventilation and carbon dioxide production; VO_2_, oxygen consumption.

### 3.2 CPET

CPET parameters of the patients are also listed in Table 2. Patients with peak VO_2_ < 13.2 mL/min/kg had higher relationship between minute ventilation and carbon dioxide production than those with peak VO_2_ ≧ 1 3.2 mL/min/kg.

By contrast, patients with peak VO_2_ < 13.2 mL/min/kg had smaller or lower values than those with peak VO_2_ ≧13.2 mL/min/kg for the following parameters: rest VO_2_ (3.2 ± 0.6 mL/min/kg vs. 3.5 ± 0.5 mL/min/kg, *p* = 0.007), anaerobic threshold (7.6 ± 1.1 mL/min/kg vs. 10.2 ± 1.2 mL/min/kg, *p* < 0.001), peak VO_2_ (9.6 ± 2.2 mL/min/kg vs. 16.1 ± 2.0 mL/min/kg, *p* < 0.001), peak heart rate (92 ± 15 bpm vs. 118 ± 15 bpm, *p* < 0.001), peak systolic blood pressure (148 ± 27 mmHg vs. 169 ± 28 mmHg, *p* < 0.001), and peak load (52 ± 22 watt vs. 92 ± 23 watt, *p* < 0.001).

### 3.3 Univariable and multivariable analyses of factors associated with peak VO_2_ < 12 mL/min/kg

Table 3 summarizes the associations between independent factors and peak VO_2_ < 12 mL/min/kg determined using univariable and multivariable analyses. Significantly associated factors in the univariable analyses were as follows: age (odds ratio [OR] 1.06, 95% confidence interval [CI] 1.02−1.11, *p* = 0.009), log NT‐proBNP (OR 1.57, 95% CI 1.06−2.33, *p* = 0.02), E/e’ (OR 1.21, 95% CI 1.08−1.35, *p* = 0.001), LVGLS (OR 1.20, 95% CI 1.05−1.38, *p* = 0.008), TAPSE (OR 0.87, 95% CI 0.78−0.98, *p* = 0.02), and DIRVF (OR 15.7, 95% CI 3.98−61.6, *p* = 0.00008). In the multivariable analysis, DIRVF was the only independent predictor of peak VO_2_ < 12 mL/min/kg (OR 6.33, 95% CI 1.28−31.1, *p* = 0.02).

**TABLE 3:** Univariable and multivariable logistic regression analyses for predicting impaired exercise capacity (peak VO_2_ < 12 mL/min/kg).

	Univariable analysis			Multivariable analysis		
Variables	OR	95% CI	*p* value	OR	95% CI	*p* value
Age	1.06	1.02–1.11	0.009	1.06	0.99–1.13	0.09
Sex	0.66	0.24–1.84	0.43	0.60	0.14–2.64	0.50
STEMI	1.28	0.45–3.46	0.65			
Multi‐vessel disease	1.64	0.64–4.18	0.30			
Hemoglobin	0.94	0.74–1.20	0.64			
Creatinine	4.11	0.91–18.7	0.07			
Maximum creatine kinase	1.00	1.00–1.00	0.19			
Log NT‐proBNP	1.57	1.06–2.33	0.02	0.86	0.50–1.49	0.60
E/A	1.32	0.43–4.04	0.63			
E/e'	1.21	1.08–1.35	0.001	1.08	0.93–1.25	0.33
LVEF	0.96	0.92–1.00	0.06			
LVGLS	1.20	1.05–1.38	0.008	1.18	0.94–1.48	0.15
IVC diameter	2.85	0.76–10.7	0.12			
IVC collapse with sniff	1.60	0.56–4.52	0.38			
TRV	0.83	0.53–1.29	0.41			
TAPSE	0.87	0.78–0.98	0.02	0.96	0.81–1.12	0.58
RV s'	0.86	0.73–1.02	0.08			
RV e'	0.92	0.76–1.12	0.42			
FAC	0.99	0.93–1.06	0.81			
DIRVF	15.7	3.98–61.6	0.00008	6.33	1.28–31.1	0.02

Abbreviations: CI, confidence interval; LVGLS, left ventricular global longitudinal strain; OR, odds ratio.

### 3.4 Univariable and Multivariable Analyses of Factors Associated With DIRVF

The results of univariable and multivariable analyses of the association between right heart hemodynamic and function‐associated parameters, along with culprit lesions that might affect them, and DIRVF are shown in Table 4. The only significantly associated factor in the univariable analysis was inferior vena cava (IVC) diameter (OR 7.83, 95% CI 1.55−39.5, *p* = 0.01). Moreover, IVC was the only independent predictor of DIRVF according to the multivariable analysis (OR 9.67, 95% CI 1.58−59.2, *p* = 0.01).

**TABLE 4:** Univariable and multivariable logistic regression analysis for determining discontinuous Doppler‐derived intrarenal venous flow.

	Univariable analysis			Multivariable analysis		
Variables	OR	95% CI	*p* value	OR	95% CI	*p* value
IVC diameter	7.83	1.55–39.5	0.01	9.67	1.58–59.2	0.01
IVC collapse with sniff	2.65	1.55–39.6	0.09	1.63	0.41–6.48	0.49
TRV	1.65	0.86–3.16	0.13	1.82	0.81–4.11	0.15
TAPSE	0.91	0.80–1.03	0.15	0.86	0.73–1.01	0.07
RV s'	0.96	0.86–1.15	0.67			
RV e'	1.03	0.82–1.28	0.82	1.19	0.91–1.58	0.21
FAC	0.97	0.90–1.05	0.50			
RCA	1.56	0.54–4.56	0.41	2.06	0.56–7.65	0.28
LAD	0.82	0.29–2.35	0.72			
LCX	0.55	0.06–4.88	0.41			
Multi‐vessel disease	0.91	0.30–2.76	0.87			

Abbreviations: CI, confidence interval; OR, odds ratio.

### 3.5 Incremental Value of Adding DIRVF and E/E’ for Predicting Peak VO_2_ < 12 mL/Min/Kg

**FIGURE 2:**
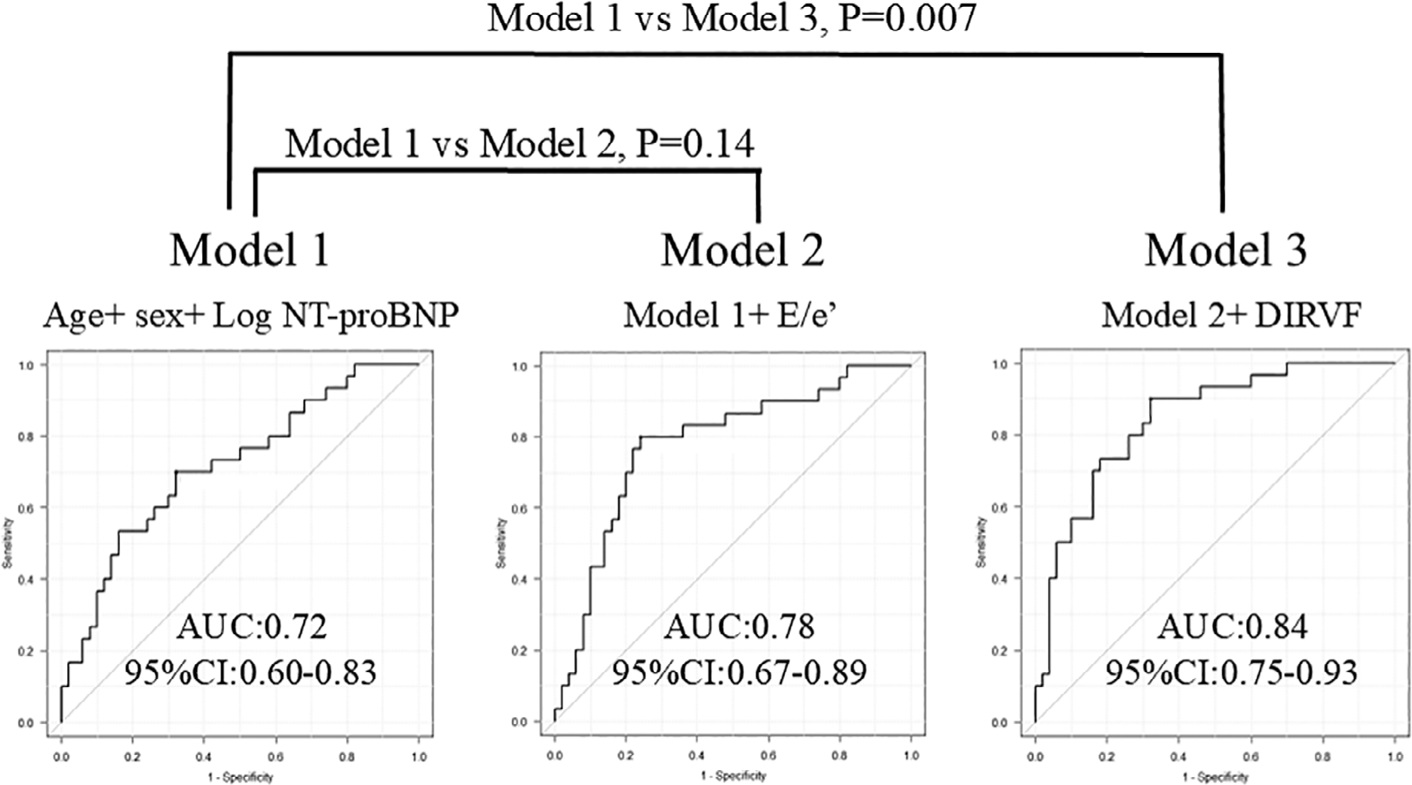
Receiver operating characteristic curve analysis using age, sex, log N‐terminal pro‐B‐type‐natriuretic peptide (NT‐proBNP) (Model 1), Model 1 plus peak early diastolic mitral inflow velocity to early diastolic velocity ratio (E/e’) (Model 2), and Model 2 plus discontinuous Doppler‐derived intrarenal venous flow (DIRVF) (Model 3). AUC, area under the curve; CI, confidence interval.

Figure 2 illustrates the incremental value of adding E/e’ and DIRVF to a model for predicting peak VO_2_ < 12 mL/min/kg, based on age, sex, and log NT‐proBNP (Model 1). The AUC obtained with Model 1 alone was 0.72 (95% CI 0.60−0.83); when E/e’ was added (Model 2), the AUC increased to 0.78 (95% CI 0.67−0.89) (Model 1 vs. Model 2, *p =* 0.14). When DIRVF was added to Model 2, the AUC was significantly higher than Model 1, increasing to 0.84 (95% CI 0.75−0.93) (Model 1 vs. Model 3, *p =* 0.007).

### 3.6 Clinical Outcome

**FIGURE 3:**
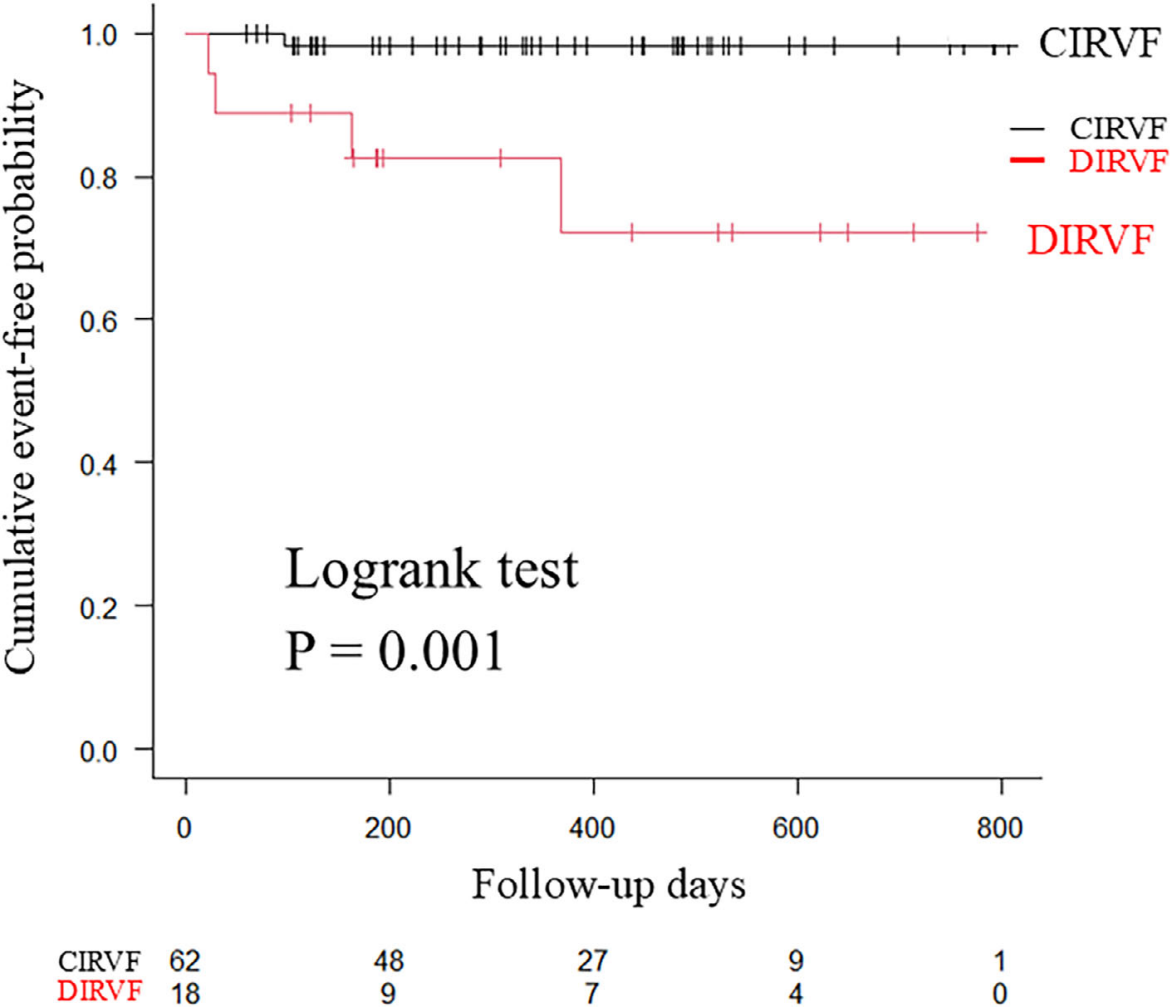
Kaplan−Meier failure curves in patients with CIRVF (black line) and those with DIRVF (red line) for composite endpoints. CIRVF, continuous Doppler‐derived intrarenal venous flow; DIRVF, discontinuous IRVF.

The median follow‐up was 366 days [189−513 days]. Composite endpoints were recorded in five patients, including one cardiovascular death and four unplanned hospitalizations for HF. The occurrence of composite endpoints was significantly higher in patients with DIRVF than in those with CIRVF (*p =* 0.001) (Figure 3).

## 4 Discussion

To the best of our knowledge, ours is the first study to investigate the efficacy of IRVF as a predictor of impaired exercise capacity and prognosis in patients with ACS. Our major findings are as follows: first, DIRVF was an independent predictor of impaired exercise capacity and poor prognosis in patients with ACS; second, addition of DIRVF to a clinical variable‐based model improved its predictive ability for impaired exercise capacity in patients with ACS.

### 4.1 IRVF

Increased central venous pressure leads to renal congestion, which is associated with the prognosis of patients with HF [1, 2]. In recent years, several reports have suggested that IRVF, which is a measure of renal congestion and intrarenal hemodynamics, is useful for predicting the prognosis of patients with HF [3, 4, 5, 6, 7]. However, no studies have examined the usefulness of IRVF in patients with ACS.

In this study, we found that DIRVF was an independent predictor of impaired exercise capacity (peak VO_2_ < 12 mL/min/kg) in patients with ACS; moreover, there was a significant difference in the occurrence of composite endpoints of cardiac events in patients with DIRVF. The prognostic results were similar to those in previous reports investigating patients with HF [3, 4, 5, 6, 7].

Previous studies have also shown that E/e’, which is a representative index of left ventricular diastolic dysfunction, is associated with exercise tolerance in patients with ACS [12, 14, 15]. In this study, E/e’ was a useful predictor of impaired exercise capacity in patients with ACS according to univariable analyses; however, multivariable analyses showed that the final strongest predictor was DIRVF.

In previous studies, echocardiography was performed in almost all cases within 1 month after onset [12, 14, 15]. The same was true in this study. It is assumed that many cases still exhibit left ventricular regional wall motion abnormalities at this stage. The presence of these abnormalities in the septal region is thought to affect the E/e’ ratio, and it is assumed that there are a certain number of cases where an increase in E/e’ is not related to an increase in left ventricular filling pressure. Previous studies have not addressed this point. We believe this is one of the reasons why E/e’ did not remain a useful parameter in the multivariable analyses.

In this study, we focused on IRVF, an indicator of right heart congestion and organ congestion. This is because systemic congestion, venous congestion associated with increased right heart pressure, and central venous pressure have attracted attention in recent years as factors that determine the prognosis of patients with HF [9]. Seo et al. demonstrated that IRVF might not only be a marker of renal congestion but also a mirror of right‐sided heart hemodynamics [7]. In this study, IVC diameter was identified as the strongest independent predictor of DIRVF. In contrast, no association was observed with tricuspid regurgitation velocity, TAPSE, early diastolic annular tissue velocity of the lateral tricuspid annulus, or ACS with the right coronary artery as the culprit lesion, all of which are thought to affect right ventricular hemodynamics and function. However, no correlation was found between the IVC itself and impaired exercise capacity in patients with ACS.

Regarding parameters of the right ventricular function or hemodynamics, it is known that each parameter is subject to various constraints [20]. For example, IVC can be influenced by the presence of positive end‐expiratory pressure. Although not included in this study, in cases managed with positive pressure ventilation management or in patients with severe chronic obstructive pulmonary disease, IVC may not accurately reflect right atrial pressure. IRVF comprehensively reflects not only IVC but also other parameters indicating right ventricular function. As a result, we believe that it may compensate for the limitations of evaluating by using IVC or TRV alone. We consider the above factors to be the reason why DIRVF ultimately remained a useful parameter in this study.

### 4.2 Ability of DIRVF Combined With E/E’ to Predict Impaired Exercise Capacity

Previous reports have found that high LV filling pressure after ACS is a predictor of poor outcomes after ACS [21] and that E/e’ is the most accurate noninvasive marker of elevated LV filling pressure [18, 22]. Moreover, E/e’ correlates with exercise capacity and prognosis in patients with ACS [12, 14, 15]. In particular, Fontes‐Carvalho et al. demonstrated that among resting diastolic function parameters, E/e’ and e’ are the strongest echocardiographic correlates of peak VO_2_ after ACS [14]. Iwahashi et al. demonstrated that E/e’ > 15 obtained 2 weeks after ACS onset was the strongest predictor of death from cardiovascular disease or unplanned hospitalization for HF. In this study, ROC analysis showed that the AUC for predicting impaired exercise capacity using basic clinical variables was 0.72; when E/e’ was added to the model, the AUC increased to 0.78; however, this was not a significant increase. By contrast, when DIRVF was added, the AUC significantly increased to 0.84. Therefore, using not only E/e’ but also DIRVF improved the predictive ability of a model for impaired exercise capacity.

We speculate that evaluating both E/e’, which indicates left heart congestion, and DIRVF, which indicates right heart congestion, allows for a more comprehensive heart congestion evaluation, resulting in better prediction of impaired exercise capacity in patients with ACS.

### 4.3 Clinical Implications

IRVF is a relatively easy parameter to acquire and analyze. In this study, acquisition and analysis of IRVF were possible in 97% of cases. We believe that renal Doppler ultrasonography can be performed without spending a lot of time by recording it from right intrarenal vein while the patient is in the left lateral position after echocardiography.

In addition, our study has shown that DIRVF is an independent predictor of impaired exercise capacity in patients with ACS, and that evaluating it together with NT‐proBNP and E/e’ improves diagnostic efficiency. For facilities that do not have a CPET equipment or do not have the time to perform CPET, we believe that acquiring IRVF can be a substitute for CPET, leading to time and cost savings.

Therefore, we recommend the acquisition of IRVF in addition to echocardiographic parameters in routine clinical practice.

### 4.4 Limitations

Our study has some limitations. First, our sample consisted of a small number of patients from a single center. Therefore, our results must be confirmed in a prospective study with a larger number of patients. Second, in this study, there were only 31 patients with peak VO_2_ less than 12 mL/min/kg, and 18 patients with DIRVF. Clinical outcome events occurred in 5 patients, resulting in a low number of events overall. We believe that the small number of cases and events has influenced both the univariable and multivariable logistic regression analyses. Moreover, the follow‐up duration was relatively short; long‐term follow‐up might yield different results.

Third, the IRVF pattern was determined through discussion between two observers (Y.S. and M.T.). Accurate intraobserver and interobserver variability could not be examined, and reproducibility of execution may not have been guaranteed in this study. We believe that reproducibility of execution should be confirmed in future researches.

Fourth, we did not perform a right heart catheterization. If we had been able to directly assess the hemodynamics of the right heart system through right heart catheterization, we believe we could have conducted a more detailed study on its relationship with exercise tolerance.

Finally, three patients were unable to exercise in this study; the average age of the group was 69 years old, and some of the subjects were elderly. Therefore, some patients terminated CPET from due to leg fatigue rather than respiratory fatigue, which may have affected their peak VO_2_ result.

## 5 Conclusions

DIRVF predicts impaired exercise capacity, which is associated with poor prognosis, in patients with ACS. We recommend the acquisition of IRVF in routine clinical practice.

## Conflicts of Interest

The authors declare no conflicts of interest.

## Ethics Statement

We obtained approval from the institutional Ethics Committee of the National Hospital Organization Kure Medical Center.

## References

[1] W. Mullens , Z. Abrahams , G. S. Francis , et al., “Importance of Venous Congestion for Worsening of Renal Function in Advanced Decompensated Heart Failure,” Journal of the American College of Cardiology 53 (2009): 589–596. 19215833 10.1016/j.jacc.2008.05.068 PMC2856960

[2] K. Damman , V. M. van Deursen , G. Navis , et al., “Increased Central Venous Pressure Is Associated With Impaired Renal Function and Mortality in a Broad Spectrum of Patients With Cardiovascular Disease,” Journal of the American College of Cardiology 53 (2009): 582–588. 19215832 10.1016/j.jacc.2008.08.080

[3] N. Iida , Y. Seo , S. Sai , et al., “Clinical Implications of Intrarenal Hemodynamic Evaluation by Doppler Ultrasonography in Heart Failure,” Journal of the American College of Cardiology: Heart Failure 4 (2016): 674–682. 10.1016/j.jchf.2016.03.016 27179835

[4] P. Nijst , P. Martens , M. Dupont , et al., “Intrarenal Flow Alterations During Transition From Euvolemia to Intravascular Volume Expansion in Heart Failure Patients,” Journal of the American College of Cardiology: Heart Failure 5 (2017): 672–681. 10.1016/j.jchf.2017.05.006 28711449

[5] A. Puzzovivo , F. Monitillo , P. Guida , et al., “Renal Venous Pattern: A New Parameter for Predicting Prognosis in Heart Failure Outpatients,” Journal of Cardiovascular Development and Disease 5 (2018): 52. 30400289 10.3390/jcdd5040052 PMC6306853

[6] F. Husain‐Syed , H. W. Birk , C. Ronco , et al., “Doppler‐Derived Renal Venous Stasis Index in the Prognosis of Right Heart Failure,” Journal of the American Heart Association 8 (2019): e013584. 31630601 10.1161/JAHA.119.013584 PMC6898799

[7] Y. Seo , N. Iida , M. Yamamoto , et al., “Doppler‐Derived Intrarenal Venous Flow Mirrors Right‐Sided Heart Hemodynamics in Patients With Cardiovascular Disease,” Circulation Journal 84 (2020): 1552–1559. 32669529 10.1253/circj.CJ-20-0332

[8] J. C. Burnett Jr. and F. G. Knox , “Renal Interstitial Pressure and Sodium Excretion During Renal Vein Contraction,” American Journal of Physiology 238 (1980): F279–282. 7377299 10.1152/ajprenal.1980.238.4.F279

[9] J. Rangaswami , V. Bhalla , J. E. A. Blair , et al., “Cardiorenal Syndrome: Classification, Pathophysiology, Diagnosis, and Treatment Strategies: A Scientific Statement From the American Heart Association,” Circulation 139 (2019): e840. 30852913 10.1161/CIR.0000000000000664

[10] L. Vanhees , R. Fagard , L. Thijs , et al., “A Prognostic Significance of Peak Exercise Capacity in Patients With Coronary Artery Disease,” Journal of the American College of Cardiology 23 (1994): 358–363. 8294687 10.1016/0735-1097(94)90420-0

[11] S. J. Keteyian , C. A. Brawner , P. D. Savage , et al., “Peak Aerobic Capacity Predicts Prognosis in Patients With Coronary Heart Disease,” American Heart Journal 156 (2008): 292–300. 18657659 10.1016/j.ahj.2008.03.017

[12] H. Tashiro , A. Tanaka , H. Ishii , et al., “Reduced Exercise Capacity and Clinical Outcomes Following Acute Myocardial Infarction,” Heart and Vessels 35 (2020): 1044–1050. 32152731 10.1007/s00380-020-01576-2

[13] D. W. Kitzman and L. Groban , “Exercise Intolerance,” Heart Failure Clinic 4 (2008): 99–115. 10.1016/j.hfc.2007.12.002 PMC2700357 18313628

[14] R. Fontes‐Carvalho , F. Sampaio , M. Teixeira , et al., “Left Ventricular Diastolic Dysfunction and E/E Ratio as the Strongest Echocardiographic Predictors of Reduced Exercise Capacity After Acute Myocardial Infarction,” Clinical Cardiology 38 (2015): 222–229. 25707582 10.1002/clc.22378 PMC6711017

[15] N. Iwahashi , K. Kimura , M. Kosuge , et al., “‘E/E’ Two Weeks After On Set Is a Powerful Predictor of Cardiac Death and Heart Failure in Patients With a First‐Time ST Elevation Acute Myocardial Infarction,” Journal of the American Society of Echocardiography 25 (2012): 1290–1298. 23200417 10.1016/j.echo.2012.09.010

[16] E. A. Amsterdam , N. K. Wenger , R. G. Brindis , et al., “AHA/ACC Guideline for the Management of Patients With Non‐ST‐Elevation Acute Coronary Syndromes: A Report of the American College of Cardiology/American Heart Association Task Force on Practice Guidelines,” Journal of the American College of Cardiology 64, no. 24 (2014): e139–228. 25260718 10.1016/j.jacc.2014.09.017

[17] M. Roffi , C. Patrono , J. P. Collet , et al., “2015 ESC Guidelines for the Management of Acute Coronary Syndromes in Patients Presenting Without Persistent ST‐Segment Elevation: Task Force for the Management of Acute Coronary Syndromes in Patients Presenting Without Persistent ST‐Segment Elevation of the European Society of Cardiology (ESC),” European Heart Journal 37, no. 3 (2016): 267–315. 26320110 10.1093/eurheartj/ehv320

[18] R. M. Lang , L. P. Badano , V. Mor‐Avi , et al., “Recommendations for Cardiac Chamber Quantification by Echocardiography in Adults: An Update From the American Society of Echocardiography and the European Association of Cardiovascular Imaging,” Journal of the American Society of Echocardiography 16 (2015): 233–270. 10.1093/ehjci/jev014 25712077

[19] M. R. Mehra , C. E. Canter , M. M. Hannan , et al., “International Society for Heart Lung Transplantation Infectious Diseases Council, International Society for Heart Lung Transplantation Pediatric Transplantation Council, International Society for Heart Lung Transplantation Heart Failure, Transplantation Council. The 2016 International Society for Heart Lung Transplantation Listing Criteria for Heart Transplantation: A 10‐Year Update,” Journal of Heart and Lung Transplantation 35 (2016): 1–23. 10.1016/j.healun.2015.10.023 26776864

[20] L. G. Rudski , W. W. Lai , J. Afilalo , et al., “Guidelines for the Echocardiographic Assessment of the Right Heart in Adults: A Report From the American Society of Echocardiography Endorsed by the European Association of Echocardiography, a Registered Branch of the European Society of Cardiology, and the Canadian Society of Echocardiography,” Journal of the American Society of Echocardiography 23 (2010): 685–713. 20620859 10.1016/j.echo.2010.05.010

[21] F. Nijland , O. Kamp , A. J. Karreman , et al., “Prognositic Implications of Restrictive Left Ventricular Filling in Acute Myocardial Infarction: A Serial Doppler Echocardiographic Study,” Journal of the American College of Cardiology 30 (1997): 1618–1624. 9385885 10.1016/s0735-1097(97)00369-0

[22] S. F. Nagueh , R. Bhatt , R. P. Vivo , et al., “Echocardiographic Evaluation of Hemodynamics in Patients With Decompensated Systolic Heart Failure,” Circulation: Cardiovascular Imaging 4 (2011): 220–227. 21398512 10.1161/CIRCIMAGING.111.963496

